# Influenza vaccination coverage of professionals working in nursing homes in France and related determinants, 2018–2019 season: a cross-sectional survey

**DOI:** 10.1186/s12889-022-13412-5

**Published:** 2022-05-25

**Authors:** Sophie Vaux, Laure Fonteneau, Anne-Gaëlle Venier, Arnaud Gautier, Sophan Soing Altrach, Pierre Parneix, Daniel Lévy-Bruhl

**Affiliations:** 1 Santé publique France (French Institute for Public Health Surveillance), Saint-Maurice, France; 2 Nouvelle Aquitaine Healthcare-Associated Infection Control Centre, Bordeaux University Hospital, Bordeaux, France

**Keywords:** Vaccination, Influenza, Nursing homes, Healthcare workers, Vaccine coverage

## Abstract

**Background:**

The burden of influenza morbidity and mortality in nursing homes (NH) is high. Vaccination of residents and professionals working in NH is the main prevention strategy. Despite recommendations, vaccination coverage among professionals is generally low.

**Methods:**

We performed a nationwide cross-sectional survey of NH using a single-stage stratified random sampling design to estimate influenza vaccination coverage in NH healthcare workers (HCW) and non-medical professionals in France during the 2018–2019 season, and to identify measures likely to increase it. For each NH, a questionnaire was completed with aggregated data by one member of the management team. A multivariate analysis was performed using a negative binomial regression.

**Results:**

Five-hundred and eighty nine NH filled in the study questionnaire (response rate: 49.5%). When considering all professionals (i.e., HCW and non-medical professionals), overall vaccination coverage was 30.6% (95%CI [28.2–33.0], range: 1.6–96.2). Overall influenza vaccination coverage in HCW was 31.9% [29.7–34.1]. It varied according to occupational category: 75.5% [69.3–81.7] for physicians, 42.9% [39.4–46.4] for nurses, 26.7% [24.5–29.0] for nursing assistants, and 34.0% [30.1–38.0] for other paramedical personnel. Vaccination coverage was higher i) in private nursing homes (RRa: 1.3, [1.1–1.5]), ii) in small nursing homes (0.9 [0.8–0.9]), iii) when vaccination was offered free of charge (1.4, [1.1–1.8]), iv) when vaccination promotion for professionals included individual (1.6 [1.1–2.1]) or collective (1.3 [1.1–1.5]) information sessions, videos or games (1.4 [1.2–1.6], v) when information on influenza vaccines was provided (1.2 [1.0–1.3], and finally, vi) when a vaccination point of contact—defined as an HCW who could provide reliable information on vaccination—was nominated within the nursing home (1.7 [1.3–2.2]).

**Conclusions:**

Urgent and innovative actions are required to increase coverage in HCW. Vaccination programmes should include free on-site vaccination and education campaigns, and particularly target nursing assistants. The results of this nationwide study provide keys for improving influenza vaccination coverage in HCW. Programmes should ensure that information on influenza vaccines is provided by a vaccination point of contact in NH using attractive media. Combining the different prevention measures proposed could increase coverage in NH nationwide by over 50%.

## Background

High rates of morbidity and mortality continue to make influenza virus infection a major public health problem [1, 2]. Influenza is particularly dangerous for at-risk patients (persons aged over 64 years, and patients with chronic disease), especially in nursing homes (NH) [3, 4]. Despite high coverage among residents in NH, outbreaks still occur in particular due to the vaccination’s limited effectiveness in elderly people [4–6]. Insufficient coverage of healthcare workers (HCW) and other professionals in contact with NH residents can introduce the virus into NH and contribute to its dissemination among residents. Studies on the effectiveness of HCW vaccination in protecting residents are difficult to implement as several biases are involved and few of the studies conducted to date have demonstrated its effectiveness. While most results suggest a protective effect, the evidence is not very strong [7–9]. Annual seasonal influenza vaccination in France is recommended for all persons over 64 years old, NH residents, HCW and professionals in contact with NH residents. The objective for vaccine coverage of HCW and professionals in contact with patients at risk is 75% [10]. National estimates of vaccination uptake by HCW working in NH were low (33.6% (95% CI: 31.9–35.4) as assessed in a survey conducted in 2009 [11], reflecting findings in other industrialized countries despite recommendations [7].

## Methods

We conducted a nationwide study during the 2018–2019 influenza season to provide updated data on NH HCW vaccination coverage in France, to identify determinants of vaccination status, and to indicate measures which appear to increase vaccination coverage in these settings.

### Study Population

The study population is HCW (physicians, nurses, nursing assistants, and other paramedical personnel) and non-medical professionals (administrative staff, cleaning staff and recreational staff) working in NH in France. All professionals are considered to be in contact with residents.

Professionals (i.e., HCW and non-medical professionals) employed for less than three months and students (medical or nursing students) were not included in the study.

The types of NH studied were medico-social institutions. Short-stay care facilities and retirement homes (i.e., with individual apartments) were not studied, as living conditions in these structures reflect those in the general community, where residents are less dependent and have fewer shared activities with other elderly persons.

The study covered all 13 regions in metropolitan France and four overseas territories (the islands of Guadeloupe and Martinique in the Caribbean Sea, French Guiana in South America, and Reunion Island in the Indian Ocean). Mayotte Island, located in the Indian Ocean, was not included due to the absence of NH in the territory.

### Sample size and randomisation

We performed a cross-sectional survey of NH using a single-stage stratified random sampling design. The sampling frame was the list of NH recorded in France’s national medico-social and healthcare institution database (FINESS) as of January 2019. FINESS is managed by the Ministry of Health. A total of 7 819 NH were recorded.

The required study sample size was calculated to ensure that estimates of influenza vaccination coverage could be made according to HCW occupation category (physicians, nurses and nursing assistants) at the national and regional levels with a precision of 5%. Considering an overall influenza vaccination coverage level of 36% (specifically 60% for physicians, 45% for nurses, 34% for nursing assistants, a design effect of 2 for nurses and nursing assistants and 1 for physicians, NH participation rate between 50 and 60%, and an α risk of 5%), we calculated that at least 80 NH needed to be solicited in each region. All NH in Corsica and in the four overseas territories were solicited, as the total number of NH in each of these areas was lower than the minimum 80.

NH were stratified by size (< 80 beds vs. ≥ 80 beds) and geographical location (17 regions). Due to a small number of nursing homes in some strata, four strata were collapsed into two to ensure a minimum number of NH for analysis. A total of 34 strata were created.

Overall, 1 189 NH were solicited to participate in the study (1 120 in metropolitan France, and 69 in the four overseas territories).

### Data collection

Data collection was conducted from May to July 2019 using a questionnaire sent by e-mail or by letter (when e-mail not available). For each NH, only aggregated data were collected. The questionnaire was completed by a member of the management team: the coordinating doctor, the director, or the nursing coordinator. Data on NH administrative and structural characteristics were collected: status (public, private), location, size (number of beds), affiliation to a hospital, presence of a care coordinator (physician), presence of a nursing coordinator presence of an expert in NH hygiene, number of professionals (medical, paramedical and non-medical) according to occupational category during the study period. The number of professionals vaccinated against influenza during the 2018–2019 season, according to occupational category, were also collected.

The following data on measures implemented to promote influenza vaccination for professionals during the 2018–2019 season within the NH were also collected: free on-site vaccination for professionals working in the NH, influenza vaccination promotion for professionals (use of posters, use of videos or games, organization of individual or collective information sessions, contents and type of information disseminated (e.g., influenza, flu vaccines, collective benefit of vaccination, individual benefit of vaccination), nomination of a point of contact for vaccination in the NH (defined as a HCW who could provide reliable information about vaccination, including for influenza), in-house analysis of structural barriers to vaccination and implementation of a vaccination action plan, existence of an in-house multidisciplinary group on vaccination, and finally, involvement of the NH director, the coordinating doctor or the nursing coordinator in the influenza vaccination campaign (if present). The questionnaire is available on line: https://www.santepubliquefrance.fr/content/download/118881/1758582.

Data were entered in a dedicated online questionnaire or were sent by regular mail or fax. Two reminders were sent to all non-respondents.

### Data analysis

The analysis was performed according to occupation category, classified into HCW (physicians, nurses, nursing assistants, and other paramedical personnel) and non-medical professionals (administrative staff, cleaning staff, educational and recreational staff). In order to assess the determinants of influenza vaccination coverage, and because of data aggregation and overdispersion, we performed univariate and multivariate analyses using a negative binomial regression. Considering the data, we dichotomized NH size into 1- 99 beds and ≥ 100 beds.

All determinants with a *p*-value < 0.2 in the univariate analysis were introduced in the multivariate model. Risk ratios (RR) and their 95% confidence intervals were used as measures of association. A p-value ≤ 0.05 was considered statistically significant.

Data analysis were performed using Stata 14.2® (StataCorp, Texas, USA). Specific sampling weights were calculated for each of the 34 strata created. All estimates were made using the “svy” command, which takes into account the sampling design and weights in all calculations (descriptive, confidence intervals, negative binomial regressions). The nursing home level effect was taken into account in analysis. Outcomes were given in percentages with their 95% confidence intervals (95% CI).

## Results

### Participation

Of the 1 189 NH invited to participate, 589 filled in the study questionnaire (response rate 49.5%) for the 2018–2019 influenza vaccination season. Thirty-one questionnaires were not included in the analysis, either because of insufficient quality of the reported data (*n* = 29) or because the NH or the region could not be identified (*n* = 2). The remaining 558 questionnaires (representing 524 NH in metropolitan France and 34 NH in the included overseas territories) constituted our study sample, reflecting 20 420 HCW (645 physicians, 3 506 nurses, 13 948 nursing assistants, 2 321 other paramedical personnel) and 10 938 non-medical professionals.

### Influenza vaccination coverage in HCW and non-medical professionals

When considering all professionals (i.e., HCW and non-medical professionals), overall influenza vaccination coverage was 30.6% (95%CI: [28.2–33.0], range: 1.6–96.2). Overall coverage in HCW was 31.9% [29.7–34.1] (Table 1). Specifically, coverage was 75.5% [69.3–81.7] for physicians, 42.9% [39.4–46.4] for nurses, 26.7% [24.5–29.0] for nursing assistants, and 34.0% [30.1–38.0] for other paramedical personnel. Overall coverage in non-medical professionals was 28.7% [25.1–32.3].

**Table 1:** Influenza vaccination coverage in healthcare workers (HCW) and non-medical professionals in nursing homes, by occupation category

	**Influenza vaccine coverage**	
	**%**	**CI95%**
**All professionals**	**30.6**	**28.2–33.0**
**Healthcare workers (HCW)**	**31.9**	**29.7–34.1**
Physicians	75.5	69.3–81.7
Nurses	42.9	39.4–46.4
Nursing assistants	26.7	24.5–29.0
Other paramedical personnel	34.0	30.1–38.0
**Non-medical professionals**^a^	28.7	25.1–32.3

^a^Administrative staff, cleaning staff, educational and recreational staff

Coverage in HCW was 32.1% [29.9–34.3] and 13.8% [6.8–20.8] in NH located in metropolitan France and overseas territories, respectively. Figures were particularly low in French Caribbean territories (Guadeloupe: 11.8% [1.9–21.6], Martinique: 7.8% [3.4–12.2]), and French Guiana: 17.5% [2.8–32.2]). In metropolitan France, coverage was 75.8% [69.5–82.1], 43.1% [39.6–46.7], 26.9% [24.6–29.2] and 34.2% [30.2–38.2] for physicians, nurses, nursing assistants and other paramedical personnel, respectively. Among non-medical professionals, influenza vaccination coverage was 28.8% [25.2–32.3].

### Measures organized for influenza vaccination of NH professionals

For the 2018–2019 influenza season, 97.9% [96.2–98.8] of NH in France proposed free on-site vaccination to their professionals (Table 2). Vaccination was organized in-house mainly by the NH care coordinator (physician) or nursing coordinator (71.2% [66.5–75.5]). Vaccination was promoted in almost all NH (99.2% [98.1–99.7]) and included posters (91.0% [88.0–93.3], collective (67.5% [63.4–71.4]) or individual (19.3% [15.8–23.2]) information sessions, and videos or games (7.8% [5.5–11.0]). Vaccination points of contact were nominated in 32.8% [28.5–37.4] of NH.

**Table 2:** Measures organized by nursing homes for influenza vaccination, 2018–2019 winter season (N: 558 nursing homes)

	**Organization of flu vaccination campaign** **Season 2018–2019**	
	**%**	[CI95%]
**Free provision of the influenza vaccine for professionals (medical, paramedical and non-medical)**	97.9	96.2–98.8
**Organization of in-house influenza vaccination**	94.3	91.9–96.0
If yes: (several choices possible)		
- by the care coordinator (physician) or nursing coordinator	71.2	66.5–75.5
- by the occupational practitioner	11.4	9.0–14.4
- by other nursing home staff (except medical or nurse coordinator)	46.6	41.6–51.6
- by mobile teams of vaccinators	5.8	4.2–8.0
**Organization of the promotion of influenza vaccination for professionals**	99.2	98.1–99.7
**If yes, how: (several choices possible)**		
- Posters	91.0	88.0–93.3
- Videos, games	7.8	5.5–11.0
- Collective information sessions	67.5	63.4–71.4
- Individual information sessions	19.3	15.8–23.2
- Point of contact for vaccination nominated within the nursing home ^a^	32.8	28.5–37.4
**If yes, with what contents: (several choices possible)**		
- Information on influenza vaccines	63.9	59.1–68.4
- Information on influenza	83.2	79.5–86.4
- Information on the collective benefits of vaccination (cocooning, organization of care)	68.6	64.2–72.8
- Information on the individual benefits of vaccination (avoid getting the flu, avoid infecting your family)	72.5	68.0–76.6
**Existence of a multidisciplinary group on vaccination**	21.6	17.9–25.8
**The director, the care coordinator (physician) or the nursing coordinator are involved and support the vaccination campaign**	89.1	85.6–91.9
**In-house analysis of structural barriers to vaccination and implementation of an action program**	30.4	26.1–35.0

^a^Healthcare worker (HCW) who can provide reliable information on vaccination (including influenza vaccination)

Promotional messages included information on influenza (83.2% [79.5–86.4], the individual benefits (avoid getting influenza, avoid infecting your family) (72.5% [68.0–76.6]), the collective benefit (cocooning, organization of care) (68.6% [64.2–72.8]), and the influenza vaccine itself (63.9% [59.1–68.4]).

Just under a third of NH (30.4% [26.1–35.0]) performed an in-house analysis of structural barriers to vaccination, and implemented a vaccination action programme.

### Determinants of influenza vaccination coverage in nursing homes HCW

In the multivariate analysis, private NH (RRa: 1.3 [1.1–1.5], *p* < 0.001), those not affiliated to a hospital (0.8 [0.7–0.9], *p* = 0.001, reference: not affiliated) those with fewer than 100 beds (0.9 [0.8–0.9], *p* < 0.001, reference: less than 100 beds), and those located in metropolitan France (0.4 [0.3–0.6], *p* < 0.001, reference: metropolitan France) were all associated with higher HCW vaccination coverage (Table 3). Higher coverage was also observed when a care coordinator (physician) was present (1.4 [1.1–1.7], *p* = 0.002), when vaccination was provided free of charge to all professionals (i.e., HCW and non-medical professionals) (1.4 [1.1–1.8], *p* = 0.004), when collective (1.3 [1.1–1.5], *p* = 0.002) and individual (1.6 [1.1–2.1], *p* = 0,006) information sessions were organized, when promotion of influenza vaccination was organized for in-house professionals with the use of video or games (1.4 [1.2–1.6], *p* < 0.001), when there was a point of contact for vaccination (1.7 [1.3–2.2], *p* < 0.001), when information was given on influenza vaccines (1.2 [1.0–1.3], *p* < 0.03), and finally, when the director, care coordinator (physician) or nursing coordinator was involved and supported the vaccination campaign (1.3 [1.0–1.5], *p* = 0.02).

**Table 3:** Influenza vaccination coverage of Healthcare workers (HCW). RR and RR adjusted (RRa) for potential determinants

	**Influenza vaccine coverage**		**Unadjusted univariate**			**Adjusted multivariate**		
	**%**	**CI95%**	**RR**	**CI95%**	***P*** value	**RRa**	**CI95%**	***P*** value
**All healthcare workers**	**31.9**	**29.7-34.1**						
**Nursing home category**								
Public	26.3	23.9-28.8	ref			ref		
** Private**	**48.7**	**43.9-53.4**	**1.8**	**1.5-2.0**	**<0.001**	**1.3**	**1.1-1.5**	**0.001**
Private non- profit	31.7	27.8-35.6	1.2	1.0-1.3	0.045	1.00	0.87-1.17	0.9
**Affiliated to a hospital **								
** Yes**	**25.5**	**22.5-28.4**	**0.7**	**0.6-0.8**	**<0.001**	**0.8**	**0.7-0.9**	**0.001**
No (or ‘I don’t know’)	36.0	33.0-38.9	ref			ref.		
**Size of nursing home (number of beds)**								
<100 beds	36.2	33.7-38.7	ref			ref.		
** >= 100 beds**	**24.6**	**21.3-28.0**	**0.7**	**0.6-0.8**	**<0.001**	**0.9**	**0.8-0.9**	**<0.001**
**Geographical area**								
Metropolitan France	32.1	29.9-34.3	ref			ref		
** Overseas territories **	**13.8**	**7.2-20.3**	**0.4**	**0.3-0.6**	**<0.001**	**0.4**	**0.3-0.6**	**<0.001**
**Presence of a care coordinator (physician)**								
** Yes**	**32.9**	**30.4-35.4**	**1.3**	**1.1-1.6**	**0.003**	**1.4**	**1.1-1.7**	**0.002**
No	26.0	21.5-30.5	ref			ref		
**Presence of a nursing coordinator **								
Yes	32.5	30.3-34.8	1.4	1.1-1.8	0.003			
No	22.1	16.3-27.9	ref					
**Hygiene expert in nursing home**								
Yes	32.0	29.2-34.8	1.1	1.0-1.2	0.2			
No	31.8	28.3-35.3	ref					
**Measures implemented in nursing home**								
**Provision of influenza vaccine free of charge for professionals**								
Yes	**32.2**	**30.0-34.4**	**1.6**	**1.1-2.4**	**0.02**	**1.4**	**1.1-1.8**	**0.004**
No	18.8	10.8-26.8	ref			ref		
**Organization of promotion of influenza vaccination for professionals**								
Yes	32.0	29.8-34.2	2.4	0.7-8.4	<0.2			
No	11.8	2.2-21.5	ref					
**Organization of influenza vaccine promotion**								
** Posters**								
Yes	31.7	29.4-34.0	1.0	0.8-1.2	0.9			
No	35.7	28.8-42.6	ref					
**Videos or games**								
Yes	**48.2**	**39.1-57.3**	**1.6**	**1.4-1.9**	**<0.001**	**1.4**	**1.2-1.6**	**<0.001**
No	30.5	28.3-32.7	ref			ref		
**Collective information sessions**								
Yes	**36.3**	**33.0-39.3**	**1.6**	**1.4-1.8**	**<0.001**	**1.3**	**1.1-1.5**	**0.002**
No	23.8	21.2-26.4	ref			ref		
**Individual information sessions**								
Yes	**39.3**	**33.6-44.9**	**1.3**	**1.1-1.5**	**0.002**	**1.6**	**1.1-2.1**	**0.006**
No	30.3	28.0-32.6	ref			ref		
**Point of contact for vaccination nominated within the nursing home **^**1)**^								
Yes	**37.4**	**32.8-42.0**	**1.3**	**1.1-1.5**	**<0.001**	**1.7**	**1.3-2.2**	**<0.001**
No	29.3	26.9-31.6	ref			ref		
**Promotion of influenza vaccination : contents**								
**Information about influenza vaccines**								
Yes	**34.7**	**31.8-37.6**	**1.3**	**1.1-1.5**	**<0.001**	**1.2**	**1.0-1.3**	**0.03**
No	27.1	23.7-30.4	ref			ref		
**Information about influenza**								
Yes	32.8	30.3-35.2	1.2	1.0-1.5	<0.05			
No	27.9	23.1-32.7	ref					
**Information about the collective benefit of vaccination**								
Yes	34.9	32.0-37.8	1.5	1.3-1.7	<0.001			
No	25.0	22.0-28.1	ref					
**Information about the individual benefit of vaccination**								
Yes	34.3	31.5-37.0	1.4	1.2-1.7	<0.001			
No	25.4	22.4-28.4	ref					
**Analysis of organizational barriers to vaccination and implementation of an action plan**								
Yes	**33.6**	**29.6-33.5**	**1.2**	**1.0-1.4**	**0.01**			
No	31.1	28.6-33.5	ref					
**In-house multidisciplinary group on vaccination**								
Yes	32.0	27.7-36.3	1.1	0.9-1.2	0.5			
No	31.9	29.4-34.4	ref					
**The director, the care coordinator (physician) or the nursing coordinator are involved and support the vaccination campaign**								
Yes	**32.9**	**30.5-35.3**	**1.5**	**1.2-1.9**	**<0.001**	**1.3**	**1.0-1.5**	**0.02**
No	23.2	18.5-27.8	ref			ref		

^1)^ Healthcare worker (HCW) who can provide reliable information on vaccination

The following variables were significantly associated with vaccination in the univariate analysis, but no longer significant in the multivariate analysis: in-house individual or collective information sessions, information on flu, analysis of structural barriers to vaccination, and implementation of a vaccination action plan.

A dose response relationship can be observed. For instance, the average vaccination coverage of HCW and non-medical professionals in NH which did not provide free influenza vaccination, had no point of contact and did not provide information on vaccines was 11.1% [5.1–17.2] (*n* = 9). In NH which provided free vaccination, had a point of contact, provided information on influenza vaccines and an action program, the average vaccination coverage was 35.2 [27.9–42.5] (*n* = 548). In NH which provided free vaccination, had a point of contact, promoted influenza vaccination, used videos or games, and provided information on influenza vaccines average vaccination coverage was 53.6% [38.9–68.4] (*n* = 16).

## Discussion

We found low influenza vaccination coverage during the 2018–2019 season in HCW (physicians, nurses, nursing assistants and other paramedical personnel) and non-medical professionals working in NH in France. Uptake varied according to occupational category. Consistent with results from other French and European studies, coverage was highest in physicians (75%) [11–13], followed by nurses, other paramedical personnel, non-medical professionals, and nursing assistants. These results showed that professionals in close contact with NH residents, and in particular nursing assistants, are insufficiently vaccinated. The 75% coverage objective is only achieved for physicians.

Coverage data for the 2018–2019 season in metropolitan France were compared with those for the 2008–2009 season (overseas data for the latter period were not available) [11]. Over the 10-year intervening period, coverage in physicians increased (2008–2009: 60.4% [CI95%: 54.9–65.8] vs. 2018–2019: 75.5% [69.3–81.7]), remained stable in nurses (45.2% [42.8–47.5] vs. 43.1% [39.6–46.7]), decreased in nursing assistants (33.7 [31.8–35.6] vs. 26.9 [24.6–29.2]), and tended to decrease in non-medical professionals (34.2 [32.0–36.3] vs. 28.8 [25.2–32.3]). Data were not collected for other paramedical personnel in 2008–2009. Accordingly, like-for-like comparison of HCW coverage cannot be made. Taking this missing data into consideration, coverage for all HCW combined remained relatively stable and low (33.6% [31.9–35.4] vs. 31.9% [29.7–34.1]. Differences in coverage according to profession became more pronounced over time. This is a particularly worrying finding, especially for nursing assistants, as they provide direct, close contact care to residents. Moreover, this finding highlights the difficulty of reaching these populations, and underlines the importance of creating tailored prevention messages for professionals working in NH.

This trend could also be explained by the controversy in France surrounding influenza vaccination in the context of the H1N1 pandemic in 2010, whereby a huge number of vaccine doses were purchased in contrast to low final uptake. This contributed to a growing reticence among the French population about vaccines in general, leading to a plurennial decrease in coverage and possibly a decrease in uptake among certain categories of professionals in NH [14]. Other perceived or real health scandals in the last decades may also have contributed to undermining the population’s confidence in vaccination.

Our study highlighted several key findings useful for policymakers to improve influenza vaccination uptake in HCW working in NH. We discuss these findings below.

### Vaccine accessibility

Providing free on-site influenza vaccination for professionals working in NH significantly improved vaccine coverage. In our study, almost 98% of NH already implemented this measure.

### In-house information sessions

The organisation of information sessions for staff in NH was associated with higher vaccine coverage whether these sessions were collective or individual.

While providing information about influenza vaccines was associated with higher HCW coverage, this was not the case for providing information about influenza, or information about the individual or collective benefits of vaccination. This result suggests that professionals wait for reliable information about influenza vaccines before deciding whether to get vaccinated or not. Studies elsewhere have shown that believing that the vaccine is effective and unlikely to cause side effects is correlated with higher uptake [15, 16].

Information sessions and providing information about influenza vaccines on vaccine uptake would very likely lead to significant improvements, since we found that less than 70% of NH in France organised such sessions for staff in the 2018–2019 influenza season or gave information about influenza vaccines.

### Media to transmit information

Although over 90% of the NH included had hung up posters to promote influenza vaccination in HCW, this medium had no influence on vaccination in this population. In contrast, using videos or games was associated with higher uptake in HCW (more than 40%). The success of these media could certainly be linked to the fact that they are better at attracting the attention of professionals because of their originality and because they foster interactive exchanges. It is important to highlight that these tools were only used in 10% of NH.

### Human contact

Having a point of contact in the NH who provided accurate vaccination information was associated with higher uptake in HCW (70%). Nevertheless, only 33% of all the NH included declared having such a person.

The points discussed above highlight that any information disseminated during a vaccination campaign must take into account the following elements: i) provide information on influenza vaccines, ii) use attractive media, and iii) be conveyed by a vaccination point of contact who can provide reliable information on vaccination. Previous studies have shown that HCW can be reluctant to search for information published by national public health institutes due to time constraints [17]. Furthermore, innovative and original information tools that can be accessed and used directly in NH, as well as train-the-trainer programmes for vaccination points of contact need to be explored. Combining these measures should make it possible to increase vaccine coverage.

In our study, coverage was also higher in NH where a care coordinator was present, and when the director, care coordinator or nurse coordinator supported and was involved in the vaccination campaign.

Higher vaccination rates were observed in private NH (vs. public NH). This finding was already observed for the 2007–2008 season in France [11]. Private nursing homes may encourage vaccination of their staff more than public ones. Furthermore, coverage was higher in small (i.e., fewer than 100 beds) NH, which reflects previous findings in France [11]. One possible reason for this is management teams in small NH are more committed to their staff’s health: falling ill may lead to HCW absenteeism; compensating for an absent colleague may be more difficult in smaller structures.

Studies on vaccine hesitancy concluded that while knowledge about efficacy and safety are key elements, societal endorsement, support from colleagues and believing that most colleagues had been vaccinated are also important [17, 18].

Finally, vaccine coverage against influenza was much lower in the four overseas territories included than in metropolitan France. Although influenza also circulates in South America and the Caribbean islands, it is possible that HCW in these territories may have felt less at risk or were more reluctant to get vaccinated for this disease. Specific studies are needed to characterise influenza vaccination hesitancy and to set up tailored vaccination campaigns in overseas territories. It is possible that uptake was underestimated for Reunion Island due to the study period, as this territory is located in the southern hemisphere.

Annual influenza vaccination is recommended for HCW worldwide, but uptake remains low in the majority of countries [19, 20]. Compulsory influenza vaccination programmes for HCW have led to uptake levels of over 95% [21–23]. Currently no country has made influenza vaccination compulsory for HCW at the national level.

Our study has limitations. First, we only collected aggregated data; individual data such as demographic characteristics, vaccine hesitancy, and knowledge about the influenza vaccine were not collected. Despite a high response rate (certainly in part thanks to the short, easy-to-fill questionnaire used), NH that did not respond to the survey may have been those where HCW vaccination initiatives were the least developed and therefore had potentially lower coverage rates. Second, the questionnaire was self-administered by NH directors, medical or nursing coordinators, and data quality cannot be verified. Finally, recall bias cannot be excluded, although we can assume that is was limited, given the relatively short time interval between the period of vaccination and the study. Estimates of influenza vaccination coverage obtained through this study were close to those observed in the surveillance of acute respiratory clusters that occurred in nursing homes during the 2018–2019 season (influenza vaccination coverage of HCW: 33%) [3]. Lastly, because of the low number of NH in overseas territories (heterogeneous islands) these data should interpreted with caution.

Influenza vaccination uptake in NH residents was not investigated in this study because it has been reported as high for many years in France [11], and was confirmed by surveillance data for the 2018–2019 season (87% uptake in NH reporting acute respiratory infection clusters) [3].

## Conclusions

This nationwide study assessed influenza vaccination coverage during the 2018–2019 season in HCW working in French NH. All types of HCW combined, coverage was low and relatively stable with respect to 2007–2008 data, with nursing assistants having the lowest coverage. Urgent and innovative actions are required to increase coverage in HCW. Vaccination programmes should include free vaccination and education campaigns, and particularly target nursing assistants. Programmes should ensure that information on influenza vaccines is provided by a vaccination point of contact in NH using attractive media. It would be interesting to conduct a similar study after the COVID crisis ends to assess whether the pandemic has modified influenza vaccine coverage in HCW.

## Data Availability

The datasets used and/or analyzed during the current study are available from the corresponding author on reasonable request.

## Abbreviations

HCW: Healthcare workers
NH: Nursing homes
RR: Risk ratios
